# *Dittrichia viscosa* L. Leaves: A Valuable Source of Bioactive Compounds with Multiple Pharmacological Effects

**DOI:** 10.3390/molecules27072108

**Published:** 2022-03-24

**Authors:** Reda Ben Mrid, Najat Bouchmaa, Imad Kabach, Zakia Zouaoui, Houda Chtibi, Mohammed El Maadoudi, Ayoub Kounnoun, Francesco Cacciola, Yassine Oulad El Majdoub, Luigi Mondello, Abdelmajid Zyad, Mohamed Nhiri

**Affiliations:** 1 Laboratory of Biochemistry and Molecular Genetics, Faculty of Sciences and Technologies of Tangier, BP 416, Tangier 90000, Morocco; ikabach@uae.ac.ma (I.K.); zakia.zouaoui@etu.uae.ac.ma (Z.Z.); mnhiri@uae.ac.ma (M.N.); 2 Institute of Biological Sciences (ISSB-P), Mohammed VI Polytechnic University (UM6P), Ben Guerir 43150, Morocco; najat.bouchmaa@um6p.ma; 3 Team of Experimental Oncology and Natural Substances, Cellular and Molecular Immuno-Pharmacology, Faculty of Science and Technology, Sultan Moulay Slimane University, Beni-Mellal 23000, Morocco; a.zyad@usms.ma; 4 Laboratory of Natural Resources and Environment, Polydisciplinary Faculty of Taza, Sidi Mohamed Ben Abdellah University of Fez, B.P. 1223 Taza-Gare, Taza 30000, Morocco; houda.chtibi@usmba.ac.ma; 5 Regional Analysis Laboratory and Research ONSSA, Tangier 93000, Morocco; mohammed.elmaadoudi@onssa.gov.ma; 6 Laboratory of Applied Biology and Pathology, Department of Biology, Faculty of Sciences of Tetouan, Abd Al-Malek Essaadi University, Tetouan 93000, Morocco; ayoub.kounnoun@uae.ac.ma; 7 Department of Biomedical, Dental, Morphological and Functional Imaging Sciences, University of Messina, 98125 Messina, Italy; 8 Department of Chemical Biological, Pharmaceutical and Environmental Sciences, University of Messina, 98168 Messina, Italy; youladelmajdoub@unime.it (Y.O.E.M.); lmondello@unime.it (L.M.); 9 Chromaleont s.r.l., c/o Department of Chemical, Biological, Pharmaceutical and Environmental Sciences, University of Messina, 98168 Messina, Italy; 10 Department of Sciences and Technologies for Human and Environment, University Campus Bio-Medico of Rome, 00128 Rome, Italy

**Keywords:** phytochemicals, antioxidants, antidiabetic, antibacterial, antiglycation, cytotoxicity

## Abstract

This work focused on the leaves of *Dittrichia viscosa*, a plant used in Mediterranean folk medicine. Compared to water extract, the methanolic extract had higher antioxidant effects. Moreover, this extract showed potent in vitro inhibitory activity against α-amylase and α-glucosidase and showed an interesting antiglycation effect. Additionally, the evaluation of the cytotoxic activity of the methanolic extract against two human breast cancer cell lines, MCF-7 and MDA-MB-468, was very promising, with no cytotoxicity towards normal cells (peripheral blood mononuclear cells (PBMCs). The antibacterial effect was also assessed and showed potent inhibitory activity against *Proteus mirabilis* and *Bacillus subtilis*. On the other hand, *Dittrichia viscosa* leaves were rich in macro-elements containing appropriate micro-elements and high levels of phenolics and flavonoids such as caffeic acid derivatives. Taken together, the results obtained in this study indicate that *Dittrichia viscosa* could constitute a valuable source of bioactive molecules and could be used either on the preventive side or for therapeutic applications without toxicity.

## 1. Introduction

Due to its geographical contrasts, Morocco offers a varied range of bioclimates, which allows for the development of rich flora composed of more than 4200 species, including more than 800 endemic species [1]. Furthermore, empirical knowledge in traditional herbal medicine has been transmitted verbally through generations and enriched thanks to a strategic geographic location. This enrichment was also linked to the constructive intermingling of Amazigh (Berber), Jewish, Saharan and Arab-Muslim, and possibly Mediterranean civilizations [2]. Additionally, herbal medicine is the most crucial type of complementary medicine [3].

*Dittrichia viscosa (D. viscosa*) is a member of the *Asteraceae* family, often used as a medicinal plant in the Mediterranean zone. In addition, numerous studies reported that *D. viscosa* has a wide range of natural compounds that can be used for alternative medicine [4].

*D. viscosa* L (Syn. *Inula viscosa* L) is known in Morocco as “Magramane”. It is a perennial species abundant in the Mediterranean areas [4]. The aerial part is used in Morocco as a decoction to treat renal diseases, diabetes, and hypertension [5]. Many investigations also reported using *D. viscosa* to treat several diseases, including rheumatic pains, cancer, bronchitis, tuberculosis, and infertility [6]. This shrub’s bioactive compounds include flavonoids, triterpenoids guaianolides, sesquiterpenes, sesquiterpene acids, lactones, and essential oils [6].

Several studies have stated that *D. viscosa* could be a source of multiple compounds that possess different pharmacological properties, such as the antioxidant effect [6,7], the cytotoxic effect [8], and wound healing activities [9]. Therefore, this work assessed the chemical profile and the antioxidant activity of the leaf extracts harvested from the northwest of Morocco. Subsequently, we examined the methanolic extract for its in vitro anti-glycation and anti-diabetic activities, as well as its cytotoxic potential on two cell lines of breast cancer. Additionally, the antibacterial effects of this methanolic extract have been evaluated.

The results obtained from this study will strengthen the knowledge about the overall medicinal properties of *D. viscosa,* highlighting another potent role of its extract, which is related to its antiglycation activity.

## 2. Results and Discussion

### 2.1. Mineral Composition of D. viscosa

Table 1 shows the mineral contents of *D. viscosa* leaves. The results obtained show that Ca and K were the most abundant macro-elements. In fact, Ca reached 19.51 g/kg, while for K, the concentration was about 14.67 g/kg. On the other hand, compared to these two elements, Mg, Na, and P had lower concentrations in the leaves of *D. viscosa;* they reached 5.93, 0.79, and 1.6 g/kg, respectively. Concerning microelements, their concentrations ranged between 439.85 mg/kg for Fe and 0.43 mg/kg for Cr. On the other hand, the concentration of Zn (187.21 mg/kg) and Mn (78.48 mg/kg) was higher in the leaves of *D. viscosa* plants. This study is the first scientific report on the mineral composition of *D. viscosa* leaves. However, in a study realized on *Inula helenium* subsp. *pseudohelenium*, belonging to the *Asteraceae* family, the composition in macro-elements was almost similar with a concentration of 19.16 g/kg for K, 14.17 g/kg for Ca, and 1.07 g/kg for Na. Notably, Mg was almost three times lower than our results (2.12 g/kg) [10]. On the other hand, these authors showed high levels of some microelements, such as Fe, Mn, and Zn; however, these concentrations remain lower than those we obtained [10].

These results show that *D. viscosa* leaves have high nutritional values considering their mineral contents, such as Ca, K, Fe, Mg, and Zn. From another viewpoint, the absence of Cd and the low concentrations of Cr and Se makes this plant a potential target for clinical use without toxicity.

### 2.2. Polyphenolic Composition of D. viscosa Leaves

**Figure 1:**
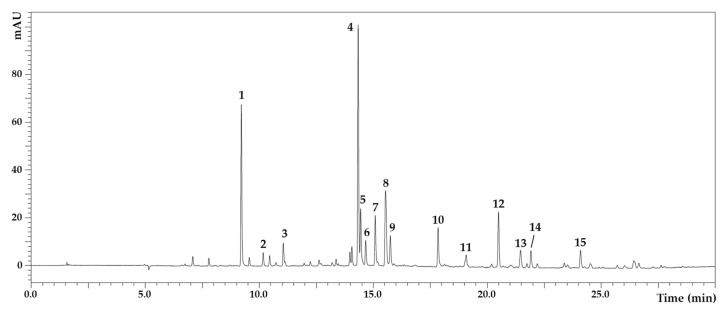
HPLC-PDA/MS chromatogram of polyphenolic compounds in *D. viscosa* methanolic extract detected at 330 nm.

Fifteen phenolic compounds were separated from the *D. viscosa* leaves (Figure 1), with details shown in Table 2. The identified compounds were caffeoylquinic acid, caffeic acid, iso-di-*O*-caffeoylquinic acid I–IV, Tri-caffeoylglucaric acid, padmatin, hispidulin, and cirsiliol. Iso-di-*O*-caffeoylquinic acid I (13.29 mg/L) was present in the highest quantity, followed by padmatin (9.00 mg/L). Caffeoylquinic acid was 8.69 mg/L, hispidulin was 4.40 mg/L, and iso-di-*O*-caffeoylquinic acid II was 3.15 mg/L.

### 2.3. Total Polyphenols and Flavonoids in D. viscosa

Phytochemical analysis showed different polyphenols and flavonoids in the methanolic and aqueous extracts (Table 3). Polyphenols and flavonoids in the methanolic extract reached 212.44 mg GAE/g dw and 123.05 mg QE/g dw, respectively. However, compared to the methanolic extract, the polyphenol and flavonoid contents were lower in the aqueous extract and reached 144.70 mg GAE /g dw and 78.89 mg QE /g dw, respectively. In a previous report, the utilization of different solvents to extract natural compounds from *D. viscosa* led to the obtention of various levels of polyphenols and flavonoids, with the highest level of polyphenol obtained by 80% ethanolic and methanolic solvents. The same report indicated that the highest concentration was obtained by butanol extraction [9]. In another study conducted by Özkan et al. [6], the contents of phenolic compounds and flavonoids of methanolic extracts obtained from the aerial part of *D. viscosa* were 107 mg GAE/g and 158.35 mg Catechol-E/g dw, respectively. In the aqueous extract, polyphenols were 59.2 mg GAE/g dw, and flavonoids were 68.5 mg CE/g dw [6]. These values are low compared to our results. These differences in phenolic and flavonoid concentration in *D. viscosa* leaves could be strongly related to environmental conditions. They can also be influenced either by the extraction method or other physiological parameters, such as the growth stage at harvest time.

### 2.4. Antioxidant Capacity of D. viscosa

Phenolic compounds and flavonoids are potent scavengers of free radicals due to their hydroxyl groups and could thus constitute up-and-coming tools for antioxidant activities. The high content of polyphenols and flavonoids that we obtained in our study for the leaves of *D. viscosa* may lead to high free radical scavenging and antioxidant activities for its extracts. To verify this, comparative assessment by in vitro tests was performed to analyze antioxidant activities of *D. viscosa* leaf extracts including DPPH^•^ and ABTS^•+^ assays, metal chelating activity, and FRAP assay (Table 3).

Irrespective of whether the methanolic or aqueous extract is used, *D. viscosa* leaves are endowed with an important DPPH radical scavenging activity. The IC_50_ for the methanolic extract was 80 µg/mL, and for the aqueous extract, it was 120 µg/mL. The capacity to quench free radicals was also determined using ABTS^+^ radicals. As for DPPH, methanolic extracts had the highest capacity to scavenge ABTS^+^ (IC_50_ = 223 µg/mL), whereas aqueous extracts exhibited the lowest activity (IC_50_ = 412 µg/mL). The ability of *D. viscosa* extracts to scavenge free radicals has already been reported in different studies. Indeed, Chahmi et al. [14] obtained an IC_50_ for DPPH of 180 µg/mL, using an ethanolic extract of *D. viscosa* from Morocco.

Moreover, in the study conducted by Gökbulut et al. [15], the water extracts of *D. viscosa* flowers exhibited the most potent DPPH radical scavenging activity among roots and leaves, using water, methanol, and ethyl acetate as solvents, the IC_50_ value of which for water extract was 0.28 mg/mL. Concerning the ABTS radical scavenging activity, few studies have been conducted. Indeed, the work conducted by Mahmoudi et al. [4] on *D. viscosa* leaves revealed that the IC_50_ of the extracts was 16.75 μg/mL for ABTS assay. The high radical scavenging activity of *D. viscosa* leaves results from the elevated phenolic compounds and flavonoids. One of the phenolic compounds isolated from *D. viscosa* leaves, 3-dicaffeoylquinic acid, was reported to have higher antioxidant capacities than Trolox and Butylated hydroxytoluene [16].

Fe^2+^ chelating activity is an essential test for estimating antioxidant capacity. The high content of this metal ion contributes to oxidative damage that may be responsible for different abnormalities in the body [17].

Consequently, the chelation of ferrous ions could represent a promising tool to avoid oxidative damage. Both methanolic and aqueous extracts exhibited chelating activity (Table 3). However, the best IC_50_ value obtained for the methanolic extract reached 0.52 mg/mL, whereas, for aqueous extraction, this value reached 2.40 mg/mL. We failed to find any previous study on the Fe^2+^ chelating activity of the *D. viscosa* leaves. However, compared to other species of the *Asteraceae* family, *D. viscosa* has a higher chelating capacity. In fact, in *Ageratum conyzoides* L., the IC_50_ value for the chelating ability of the methanol extracts reached 1.23 mg/mL, while this value was higher for the aqueous extract and reached 1.7 mg/mL [18].

FRAP assay is a well-known test that allows for the determination of the reducing capacity of active materials by depicting their electron-donating ability [19]. In this work, the FRAP assay of the methanolic extract displayed the highest antioxidant capacity with 944.19 mg AAE/g dw, while for the aqueous extracts, it was about 659.441 mg AAE/g dw. Unfortunately, only one study has evaluated the reducing power of *D. viscosa* extracts. In this study, Rhimi et al. [9] reported a value of 296.42 mg TE/g dw, three times lower than the result we obtained for the methanolic extract of *D. viscosa* leaves. This difference may be attributed either to the species, the extraction method, or the plant’s geographical environments and the development stage [20].

### 2.5. Inhibition of D. viscosa Leaves on Non-Enzymatic Glycation Process

**Figure 2:**
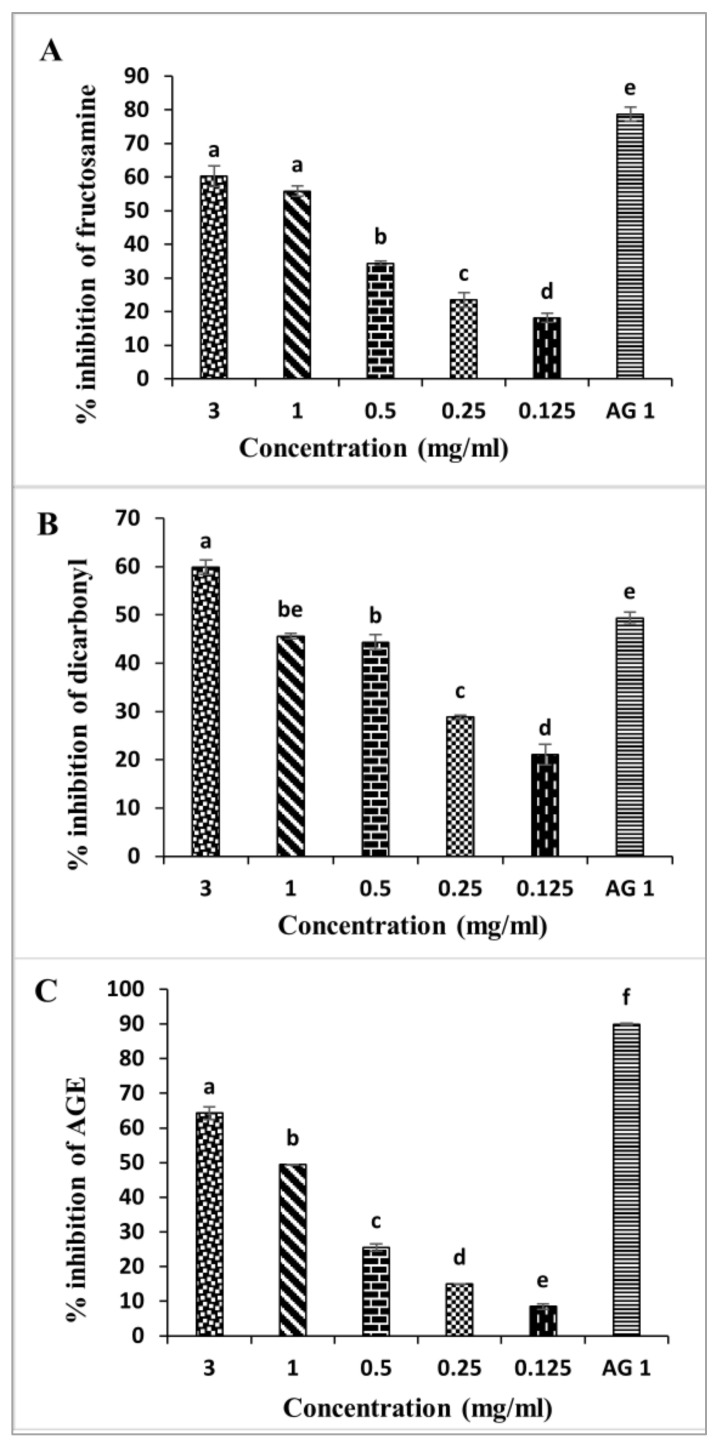
The percentage inhibition of *D. viscosa* leaf extract and aminoguanidine on (**A**) Fructosamine. (**B**) Dicarbonyl compounds. (**C**) Fluorescent AGE formation. In BSA/fructose system. Each value represents the mean of three replicates. Bars represent the standard error. Different letters indicate significant differences among treatments at *p* < 0.05.

Protein glycation is a chemical process between an amino acid or even free amino acids and reducing sugars [21]. This post-translational modification leads to various chemical by-products known as advanced glycation end products (AGEs). These irreversible molecules were reported to be implicated in many aspects of life, such as aging, and to inducing pathological complications, especially diabetes complications and different types of cancers [22]. Therefore, extracts of *D. viscosa* leaves were assessed for their abilities to inhibit the non-enzymatic glycation process following the so-called BSA assay (Figure 2).

At early glycation stages, Schiff’s bases are produced. These unstable compounds are transformed into Amadori products such as fructosamine [21]. Therefore, developing strategies to reduce fructosamine was a promising solution to delay diabetes complications [23]. The fructosamine inhibitory activity is shown in Figure 2A. Fructose-induced glycated BSA was associated with significant inhibition of fructosamine formation after the four weeks of the study. Interestingly, *D. viscosa* extract inhibits fructosamine formation at the different concentrations (0.125–3 mg/mL), with the highest inhibition observed when using 3 mg/mL of the extract. This result indicates that *D. viscosa* extract has a remarkable effect on the inhibition of fructosamine formation.

During the glycation process, the dicarbonyl intermediate is generated. This element could be more reactive than glucose. It was also reported by Thornalley [24] that the evaluation of these compounds may reflect the glycation reaction process. Therefore, to prevent and manage the complications of diabetes, inhibitors which can reduce the glycation process are extremely valuable [25]. The effects of *D. viscosa* extract on the inhibition of dicarbonyl compounds are shown in Figure 2B. The *D. viscosa* extract exhibited significant inhibitory activity on dicarbonyl compounds. Based on the results obtained, *D. viscosa* leaves could significantly reduce dicarbonyl compounds. The decrease in these compounds was dose-dependent, with the highest inhibitory activity recorded at 3 mg/mL and the lowest one at 0.125 mg/mL. The highest concentration (3 mg/mL) reduced dicarbonyl compounds by 59.82% after four weeks of incubation. By comparing these results to the positive control, aminoguanidine, we showed that at 1 mg/mL, this compound exhibited an inhibition rate of 49.34%.

The extracts of *D. viscosa* leaves were also tested with a BSA assay to assess whether they could decrease AGE formation. A concentration ranging from 0.125 to 3 mg/mL revealed a dose-dependent anti-glycation activity of the *D. viscosa* extracts (Figure 2C). At 3 mg/mL, AGE formation was reduced by 64.22% after fourweeks of incubation. For aminoguanidine, a 1 mg/mL concentration led to 89.89% inhibition of AGE formation.

To the best of our knowledge, extracts of *D. viscosa* leaves have not yet been examined for antiglycation activities. Therefore, our study on the effects of *D. viscosa* methanolic extract on glycation inhibition adds significant further information to current data about the possibility of using medicinal herbs to alleviate diabetes complications.

In this work, we showed that *D. viscosa* extracts had significant effects on the different steps of protein glycation. These effects could result from the chemical composition of the leaves of *D. viscosa*. Moreover, the potent activity of these extracts against free radicals could be one of the major mechanisms of prevention of AGE formation, as already stated in the study of Wu et al. [26]. Indeed, a recent work highlighted that those polyphenols in some edible plants ensure protective effects against protein glycation. Furthermore, hispidulin, a naturally occurring flavonoid found in *D. viscosa*, was reported to potentially inhibit nonoxidative advanced glycation end products [27]. From all the above, we may hypothesize that *D. viscosa* methanolic extracts may induce inhibition of glycation through different pathways. The natural compounds from *D. viscosa* could block early glycation products, and they can also act on intermediate products such as dicarbonyl and cause their depletion, and, finally, they can act on the final products of the process glycation and reduce their formation. 

Moreover, following the Maillard reaction, different pathways are activated in macrophages, adipocytes, endothelial cells, and other cell types after the fixation of the AGEs produced on a receptor for advanced glycation end products (RAGEs). These reactions lead to prolonged inflammatory and oxidative stress and increased synthesis of ROS. In our study, the effect of *D. viscosa* extract has shown a potent power in reducing free radicals, which explains its protective effect against AGE products.

### 2.6. In Vitro Study of Antidiabetic Activities of D. viscosa Leaf Extracts

**Figure 3:**
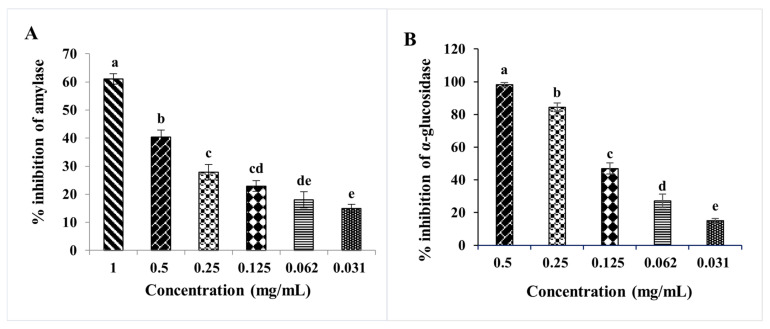
Percentage of α-amylase inhibition (**A**) and α-glucosidase inhibition (**B**) versus different concentrations of *D. viscosa* leaf extract. Each value represents the mean of three replicates. Bars represent the standard error. Different letters indicate significant differences among treatments at *p* < 0.05.

An imbalance between insulin secretion and blood glucose levels is a hallmark of type 2 diabetes. Thus, regulating the levels of sugars in plasma is crucial for delaying or avoiding Type 2 diabetes [28]. One of the most adopted methods to reduce hyperglycemia is inhibition or reducing carbohydrate absorption. Hyperglycemia reduction could be achieved by decreasing the activities of digestive enzymes such as α-amylase and α-glucosidase [29]. Indeed, acarbose is a well-known antidiabetic drug with potent inhibitory activities on *α*-amylase and *α*-glucosidase. However, as with all drugs, acarbose presents some side effects related mainly to liver toxicity [29]. Therefore, researchers have become more interested in natural bioactive molecules with α -amylase and α-glucosidase inhibition activity and with low or no side effects. Hopefully, many natural compounds have proved their effectiveness in inhibiting these two enzymes. From our side, we showed that extracts from the leaves of *D. viscosa* were also able to inhibit α-glucosidase and α-amylase activities (Figure 3). Indeed, *D. viscosa* leaves presented a high effect on both enzymes. In terms of IC_50_ (Table 4), the inhibitory effect of α-amylase was 1.381 mg/mL, while acarbose inhibitory concentration was 0.046 mg/mL. The IC_50_ values for anti-glucosidase was 0.118 mg/mL, which is significantly effective compared to acarbose IC_50_ value (0.329 mg/mL). Few studies reported the inhibitory activity of *D. viscosa* on α-amylase and α-glucosidase. Asraoui et al. [7] showed that the *D. viscosa* leaves have an important inhibitory effect against α-glucosidase with an IC_50_ of 0.0653 mg/mL and an inhibitory effect against α-amylase of 27% at 1 mg/mL.

### 2.7. Methanolic Extract Cytotoxicity of D. viscosa on MCF-7 and MDA-MB-468 Cells

**Figure 4:**
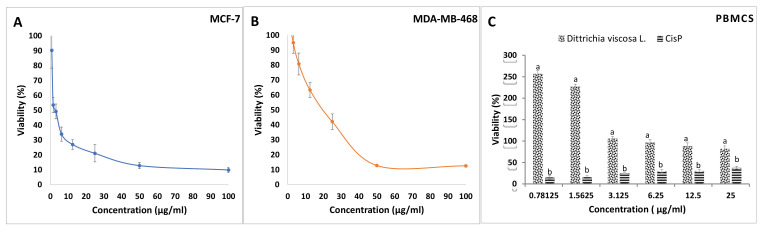
The cytotoxicity effect of *D. viscosa* leaf methanolic extracts at different concentrations for 48 h. (**A**) The cytotoxicity against the human luminal breast adenocarcinoma MCF-7 cell line. (**B**) The cytotoxicity effect against the human triple-negative breast cancer MDA-MB-468 cell line. (**C**) The viability towards the human’s peripheral blood mononuclear cells (PBMCs). Each value represents the mean ± standard deviation of three independent replicates. Different letters indicate significant differences (*p* < 0.05) within the same concentration.

An IC_50_ value of proliferation of less than 20 µg/mL is considered the threshold for a crude extract to be cytotoxic [30]. Several studies have reported cytotoxic activities of *D. viscosa* extracts against different human cancer cell lines such as cervical carcinoma cell lines (SiHa and HeLa) [31,32]. As indicated in Figure 4B, the effect of methanolic extract of *D. viscosa* was dose-dependent. Moreover, the effects on both cell lines, MCF-7, and MDA-MB-468 cell lines, were not the same. The smallest IC_50_ was obtained for MCF-7 cells after treatment with 2.75 µg/mL of the methanolic extract of *D. viscosa*. For MDA-MB-468, this value was about 20.43 µg/mL (Table 5) as MDA-MB-468 is known to be a triple-negative breast cancer cell line with the characteristics of a negative expression of progesterone receptor (PR), estrogen receptor (ER), and human epidermal growth factor receptor-2 (Her-2), while the MCF-7 line positively expresses ER [31]. Our data indicate that the bioactive compounds isolated by methanolic extraction from the leaves of *D. viscosa* could potentially prevent the growth of different types of breast cancer cells. However, more studies should confirm this result and determine the molecular mechanisms responsible for the observed effects.

In the study conducted by Merghoub et al. [32] on HeLa and SiHa cells, the authors provided evidence that the hexane and dichloromethane extracts of *D. viscosa* affected telomerase machinery and induced apoptosis. In another study, Özkan et al. [6] tested methanolic and aqueous extracts of D. *viscosa* on MCF-7 and a glioblastoma cell line (T98-G) and indicated a better antiproliferative activity for the methanolic extract compared to the aqueous extracts. However, compared to our results, the IC_50_ value obtained against MCF-7 was 179.5 µg/mL, which is very high compared to the IC_50_ we obtained. The difference between results can be associated with different parameters related to the plant’s growth stage and pedoclimatic conditions. Indeed, the chemotype of the extract is very important since the secondary metabolism can vary within the same species depending on the ecosystem (such as altitude, sunshine, biotope), although their morphology and genetics are not substantially affected modified.

In the current study, the cytotoxicity regarding normal cells was evaluated. In addition, we also assessed the cytotoxic effects of *D. viscosa* leaf extract on human peripheral blood mononuclear cells (PBMCs) (Figure 4C). Therefore, as indicated in Table 4, the cytotoxicity evaluation of *D. viscosa* leaves methanolic extract against PBMCs indicated an IC_50_ > 50 µg/mL. As expected, according to the national cancer institute (NCI), *D. viscosa* methanolic extract has shown no cytotoxic effect on normal cells. Therefore, the results issued from this study support the high selective killing abilities of *D. viscosa* methanolic extracts on the tested cancerous cells (MDA-MB-468 and MCF7). Additionally, viscosa leaf extract strongly promoted the proliferation of PBMCs, which could be explained by an immune stimulant effect of this extract.

### 2.8. Antibacterial Activity of D. viscosa L. Leaf Extracts

The antimicrobial effect of *D. viscosa* L. leaf extract against different microorganisms belonging to Gram (−) and Gram (+) was quantitatively and qualitatively proved by the diameter of the zone of inhibition, in addition to the minimal inhibitory concentration (MIC) results (Table 6). According to the in vitro test, our results show that the extract of *D. viscosa* L. leaves exhibited antimicrobial activity against two bacteria tested with a variable degree. Indeed, the most sensitive bacterium was *Proteus mirabilis*, with a diameter inhibitory zone value of 11.3 ± 0.6 mm with a MIC equal to 6.79 ± 0.39E^−17^, followed by *Bacillus subtilis* (10.3 ± 0.6 mm; MIC = 1.56 ± 2.72E^−16^). On the other hand, *Staphylococcus aureus, Pseudomonas aeruginosa,* and *Escherichia coli* are not sensitive to all extracts.

The study performed by Mssillou et al. [33] on the antimicrobial effect of ethanolic and acetonic extracts of *D. viscosa* against nosocomial infections caused by *Escherichia coli, Bacillus subtilis,* and *Staphylococcus aureus* appears more efficient compared to our study. An explanation for these results could be linked to extraction solvent and/or the resistance of the used bacterial strains [33]. In the present study, *D. viscosa* showed high levels of flavonoids and polyphenols such as hispidulin, cirsiliol, and caffeic acid derivatives. These polyphenols were reported to have potent antimicrobial activities, mainly through their action on the lipid membrane and the membrane’s permeability, destabilizing the cytoplasmic membrane or disrupting the ions’ transport [34,35].

## 3. Materials and Methods

### 3.1. Plant Material and Extraction

Leaves from *Dittrichia viscosa* plants were harvested in January 2018 in the suburbs of Taza, Morocco. A voucher specimen (No. 1-130832) was prepared, authenticated by Dr. Boulli Abdelali (botanist, Sultan Moulay Slimane university), and deposited at the Laboratory of Biochemistry and Molecular Genetics, Faculty of Sciences and Technologies, Tangier (Morocco). Using distilled water (dH_2_O), the leaves were well washed, dried, and powdered, as reported by Kabach et al. [36].

The extraction of the plant material was conducted at room temperature (RT) and in dark conditions with continuous shaking for 24 h, using either dH_2_O or methanol 80%. After filtration with the Whatman filter, extracts were centrifuged at 5000× *g* for 10 min. The aqueous and methanol solvents were then dried as a powder in an incubator at 40 °C, taking 7 and 3 days, respectively. The final yields were 17.9% for aqueous extracts and 14.2% for methanolic extracts.

### 3.2. Mineral Composition Using Inductively Coupled Plasma–Atomic Emission Spectroscopy

The mineral contents were investigated using inductively coupled plasma-atomic emission spectroscopy analysis, as described in our previous work [36], using 0.5 g of *D. viscosa* leaf powder. The spectrometer Optima 8000 ICP-OES was used for measuring the metal element concentrations.

### 3.3. HPLC-DAD-ESI/MS Analysis

HPLC-DAD-ESI/MS analysis of polyphenolic compounds from the methanolic extract of *D. viscosa* was conducted on a liquid chromatography system (Shimadzu, Kyoto, Japan). The same protocol of separation and quantification has already been published in the work of Asraoui et al. [7].

### 3.4. Total Phenolic Content

To measure the total phenolic contents, we referred to the method of Folin–Ciocalteu after some slight modifications, as reported by Kabach et al. [36]. Briefly, 0.1 mL from the D. viscosa extracts was added to 0.4 mL of the Folin–Ciocalteu reagent. Then, 1 mL of Na_2_CO_3_ (prepared at a concentration of 7%) and 0.1 mL of dH_2_O were added to this mixture. A final step of incubation in the dark for 30 min was followed by measuring absorbance at 725 nm. Finally, we used a gallic acid calibration curve to quantify the total phenolic contents.

### 3.5. Total Flavonoid Content

The protocol for determining flavonoid contents was the same as Maria et al. [37]. Briefly, to 40 µL of each sample of the *D. viscosa* extracts, 10 µL of 1 M of acetate potassium and 10% aluminum chloride were added. After mixing, a volume of 100 µL of methanol at 50% was added, and the final volume was then made up to 400 µL using dH_2_O. Absorbances were read at 415 nm, and quercetin was used as standard.

### 3.6. DPPH Radical Scavenging Assay

The radical scavenging ability of aqueous and methanolic extracts of *D. viscosa* leaves was determined using DPPH (2,2-diphenyl-1-picrylhydrazyl), as reported in the study of Ben Mrid et al. [20] with some modifications. Indeed, 150 µL of freshly prepared solution of DPPH was mixed with 50 µL from each extract. After mixing, the solutions were kept in the dark for 30 min at room temperature. Then, the absorbance was measured in a spectrophotometer at 517 nm. The equation below has been used to determine the scavenging activity of the extracts on DPPH:

% Scavenging effect = [(A_DPPH_ − A_S_)/A_DPPH_] × 100

A_S_ refers to the sample absorbance; A_DPPH_ is the absorbance of the DPPH solution.

### 3.7. Radical Scavenging Effect against Acid 2,2′-Azino-bis(3-éthylbenzothiazoline-6-sulphonic) (ABTS^+^)

The radical scavenging effect of the methanolic and aqueous extracts obtained from *D. viscosa* leaves was assessed against the radical ABTS^+^ described by Ben Mrid et al. [20]. From a diluted solution of ABTS^+^, 185 µL was taken up and mixed with 15 µL of each sample. The mixtures were kept for 10 min before measuring the absorbance at 734. The following equation has been used to determine radical scavenging activity:

%Scavenging activity = [(A_ABTS_^+^ − A_S_)/A_ABTS_^+^] × 100

A_S_ and A_ABTS_^+^ refer to the absorbance of the sample and ABTS^+^ solution, respectively.

### 3.8. Metal Chelating Activity

This activity was determined for the methanolic, and aqueous extracts of *D. viscosa* leaves using the protocol of Bouchmaa et al. [23]. In glass tubes, 800 µL of increasing concentrations of each sample was added to 10 µL of 0.6 mM FeCl_2_. After vigorous mixing, the tubes were maintained at RT for 10 min. After that, 50 µL of ferrozine (5 mM) was added to start the reaction. The absorbance was determined after 10 min, at 562 nm, and the IC_50_ was calculated.

### 3.9. Reducing Power Assay (FRAP)

The FRAP assay was conducted to determine the reducing power of the *D. viscosa* extracts, using the protocol described by Oyaizu et al. [5]. In glass tubes, 200 µL of the sample extract was added to 500 µL phosphate buffer (0.2 M, pH 6.6) and 500 µL of potassium ferricyanide (1%). After 20 min at 50 °C, 500 µL of trichloroacetic acid (10%) was added to each tube, and the mixtures were centrifuged at 1500× *g* for 10 min. Thereafter, 500 µL from the supernatants were added to 500 µL of dH_2_O and 0.1 mL of FeCl_3_ (0.1%). Ascorbic acid was used as a standard, and the absorbance of each sample was determined at 700 nm.

### 3.10. Antiglycation Activities

Glycated bovine serum albumin (BSA) formation was achieved following a previous method with some modifications [21]. First, Fructose was prepared using phosphate buffer solution (PBS, 100 mM, pH 7.4) to form a 0.5 M solution. Next, BSA (10 mg/mL) was added to a fructose solution and incubated for four weeks at 37 °C. Additionally, 0.02% sodium azide was used to inhibit the microorganism’s development. Plant extracts were also prepared in PBS before adding to the mixed reactions. Advanced glycation end products (AGEs) were quantified by a spectrofluorometer (excitation wavelength: 355 nm; emission wavelength: 460 nm). In this study, a positive control consisting of aminoguanidine was used. The inhibition rates of the AGE formation were measured following the equation:

$$\mathrm{Inhibition}\;\left(\%\right)=\left[1-\frac{\left(\mathrm{FLs}-\mathrm{FLsb}\right)}{\left(\mathrm{FLc}-\mathrm{FLcb}\right)}\right]\times 100$$

where FLs represents the fluorescence intensity of the mixture, FLsb corresponds to the fluorescence intensity of the sample blank (without fructose), FLc is the fluorescence intensity of the control mixture, and FLcb refers to the fluorescence intensity of the control blank mixture.

#### 3.10.1. Measurement of Fructosamine Inhibition

The fructosamine assay was tested after four weeks of incubation. Fructosamine reduces NBT and produces coloration, absorbing at 530 nm [38]. Briefly, 40 µL of the glycated materials was taken up, and 160 µL of 0.3 mM of NBT was added. The absorbance of the mixture was determined at 530 nm after an incubation step at 37 °C for 30 min.

Inhibition of fructosamine by *Dittrichia viscosa* (%) = (A_i_ − A_j_)/A_i_ × 100,

where A_i_ represents the absorbent value of the control mixture and A_j_ corresponds to the glycation system in the presence of inhibitor.

#### 3.10.2. Measurement of Dicarbonyl Inhibition

The capacity of dicarbonyl compounds inhibition was evaluated as described by Huang et al. [39]. First, 20 µL of glycated samples was added to 10 µL of 500 mM Girard-T and 170 µL of sodium formate buffer (0.5 M, pH 2.9). The incubation step was for 60 min at RT followed by a measurement of the absorbance at 290 nm. The method for calculating the inhibition rate of *D. viscosa* is similar to the method for measuring fructosamine inhibition.

### 3.11. Antidiabetic Enzymatic Assays

#### 3.11.1. α-Amylase Inhibition Assay

The α-amylase inhibition potential of *D. viscosa* extracts was assessed following a modified protocol described by Dong et al. [40]. First, one hundred microliters from each sample solution were premixed with 100 µL of 0.1 U/mL α-amylase solutions prepared in PBS and kept at 37 °C for 30 min. After that, 100 µL of starch solution (0.25%) prepared with PBS (pH 6.9) was added. The mixtures were maintained at 37 °C for 30 min before adding 200 µL of the 3,5-dinitrosalicylic acid reagent composed of a mixture of sodium potassium tartrate (12%) and 3,5-dinitrosalicylic acid (1%). The next step consisted of boiling the samples for 5 min, and after cooling the temperature down (at RT), a spectrophotometer was used to measure the absorbance at 540 nm.

The percentage of inhibition was measured following the equation:

$$\mathrm{Inhibition}\;\left(\%\right)=\left[\frac{\left(\mathrm{Ac}-\mathrm{Acb}\right)-\left(\mathrm{As}-\mathrm{Asb}\right)}{\left(\mathrm{Ac}-\mathrm{Acb}\right)}\right]\times 100,$$

where Ac is the absorbance of the control composed of the buffer and the enzyme; Acb refers to the blank control composed of the buffer only; As and 𝐴sb correspond to the absorbance of the sample and the sample blank, respectively.

#### 3.11.2. α-Glucosidase Inhibitory Assay

The determination of inhibitory activity against α-glucosidase (from yeast) was measured following the method published by Kee et al. [41] and following the modifications reported in [7].

### 3.12. Cytotoxic Activity

#### 3.12.1. Cell Culture

Two human breast adenocarcinomas cell lines came from the Curie Institute (Translational Research Department, Breast Cancer Biology Team, Paris, France). They were subsequently obtained from the parent cultures of the laboratory of Experimental Oncology and Natural Substances of the Faculty of Science and Technology of Sultan Moulay Slimane University, Beni Mellal. Two different subtypes of cancer cell lines, the MCF-7, representing the luminal subtype of breast cancer, and MDA-MB-468, representing the triple-negative subtype, were grown in a culture medium composed of RPMI-1640, to which fetal bovine serum, at a concentration of 5%, L-glutamine at a concentration of 0.2%, and penicillin G-streptomycin at a concentration of 1% were added. The incubator has been settled at 5% CO_2_ and 37 °C.

#### 3.12.2. MTT Assay against Tumor Cells

Approximately 10^5^ and 7 × 10^4^ cells per well for MCF-7 and MDA-MB-468, respectively, were seeded in 96-well microplates that contained 0.1 mL of complete medium to undergo an in vitro cytotoxic screening as previously described in our previous works [42,43]. Cytotoxicity analysis was confirmed for 48 h in three independent experiments using the MTT assay.

#### 3.12.3. MTT Assay towards Normal Cells (“PBMCs”)

PBMCs were considered normal cells in oncology studies conducted by Bouchmaa et al. [23,42,43,44], as they came from healthy donors. So, according to the protocol of Bouchmaa et al. [44], and under the supervision of an ethical committee for Biomedical Research of Rabat “reference number IORG0006594”, blood samples were collected from healthy donors in heparinized tubes, and the peripheral blood mononuclear cells (PBMCs) were separated using standard Ficoll-hypaque density centrifugation. The cytotoxic effect was evaluated under the same conditions and concentrations previously described for tumor cells using MTT assay.

### 3.13. Antibacterial Activity

#### 3.13.1. Microorganisms and Growth Conditions

The bacterial pathogen strains used for this study were for the Gram-negative *Pseudomonas aeruginosa* CECT-118, *Escherichia coli* K12, and *Proteus mirabilis,* and the Gram-positive *Bacillus subtilis* DSM 6633 and *Staphylococcus aureus* CECT 976. These strains were cultivated in Mueller–Hinton agar at 37 °C and used for the antibacterial tests at 10^6^ CFU/mL.

#### 3.13.2. Agar Disc Diffusion Method and Minimum Inhibitory Concentration/Minimum Bactericidal Concentration (MIC/MBC) Determination

The antibacterial activity was determined as previously described [45]. The concentration of the extracts was 50 mg/mL. Dimethyl sulfoxide and Gentamicin were used as negative and positive controls, respectively. For the MIC/MBC, the protocol of Gulluce et al. [46] was followed.

### 3.14. Statistical Analysis

The used results represent means values ± standard deviation (SD). All statistics were conducted using impaired two-tailed Student’s *t*-test and one-way ANOVA or Tukey’s multiple comparison test for inter-group and intra-group comparisons, respectively, to determine the statistical significance results (*p* < 0.05), using version 18 of SPSS statistics.

## 4. Conclusions

This work reported a chemical characterization of *D. viscosa*, a plant used in Mediterranean folk medicine for centuries. Methanolic and aqueous extracts of *D. viscosa* leaves showed potent free radical scavenging and antioxidant activities, using different tests, DPPH^+^ and ABTS^+^ assays, FRAP, and ferrous ion (Fe^2+^) chelating activity. In addition, the methanolic extract showed higher levels of polyphenols and higher antioxidant abilities. For this reason, the methanolic extract was used to assess its antidiabetic, antiglycation, and cytotoxicity effect on two human breast cancer cell lines. It was proved that *D. viscosa* extracts inhibited not only α-amylase and α-glucosidase but also advanced glycation end-product formation, which would help avoid diabetes complications.

On the other hand, *D. viscosa* methanolic extract exhibited a high antibacterial effect against *Proteus mirabilis* and *Bacillus subtilis*. In addition, cytotoxic activity revealed strong cytotoxic effects against both cell lines MCF-7 and MDA-MB-468 in a dose-dependent manner. However, the cytotoxic effect was more significant against the MCF-7 cell line. Moreover, *D. viscosa* leaf extracts do not show cytotoxic activity towards normal cells (PBMCs).

Based on the results, *D. viscosa* could represent a promising source of bioactive compounds considering its effectiveness against diabetes, glycation, cancer, and resistant bacteria. Furthermore, this plant could also be used on the preventive side as it contains no Cd and low concentrations of Cr and Se.

## Figures and Tables

**Table 1:** Mineral content levels in leaves of *D. viscosa*.

Mineral Content mg/kg dw	*D. viscosa* Leaves
Macroelements	
Ca	19,511.82 ± 822.77
K	14,671.00 ± 1582.50
Mg	5932.00 ± 1043.69
Na	792.62 ± 79.68
P	1601.6 ± 357.23
Microelements	
Co	0.224 ± 0.04
Fe	439.85 ± 61.87
Mn	78.48 ± 20.73
Zn	187.21 ± 24.03
Cr	0.43 ± 0.08
Cu	12.69 ± 2.93
Se	4.2 ± 1.09
Heavy metals	
Cd	ND

Values are expressed as the mean ± S.D. ND: not detected.

**Table 2:** Characterization of phenolic compounds of *D. viscosa* extract by HPLC-PDA/ESI-MS. Column: Ascentis Express C_18_, 15 cm × 4.6 mm, 2.7 μm d.p. (ESI, negative ionization mode; when observed, secondary fragment ions are reported).

N	Tentative Identification	t_R_ (min)	UV_max_ (nm)	[M-H]^−^	Extract (mg/L)	Employed Standard for Quantification	References
1	caffeoylquinic acid	9.22	326	353, 191	8.69 ± 0.03	Caffeic acid	[6,10]
2	caffeic acid	10.17	323	179	0.17 ± 0.02	Caffeic acid	[6,10]
3	di-*O*-caffeoylquinic acid	11.06	323	515, 353	0.60 ± 0.01	Caffeic acid	[6,11]
4	iso-di-*O*-caffeoylquinic acid I	14.33	328	515, 353	13.29 ± 0.06	Caffeic acid	[6,11]
5	iso-di-*O*-caffeoylquinic acid II	14.45	328	515, 353	3.15 ± 0.08	Caffeic acid	[6,11]
6	iso-di-*O*-caffeoylquinic acid III	14.66	327	515, 353	0.88 ± 0.01	Caffeic acid	[6,11]
7	iso-di-*O*-caffeoylquinic acid IV	15.08	327	515, 353	2.35 ± 0.08	Caffeic acid	[6,11]
8	unknown	15.54	328	695, 405	-	-	-
9	Tri-caffeoylglucaric acid	15.75	328	695, 371	1.41 ± 0.01	Caffeic acid	[6,11]
10	unknown	17.84	328	428	-	-	-
11	padmatin	19.08	288	317	9.00 ± 0.30	Naringin	[6,11]
12	hispidulin	20.49	334	299	4.40 ± 0.05	Apigenin	[3,6,11,12,13]
13	unknown	21.45	290	417	-	-	-
14	cirsiliol	21.92	358	329	1.89 ± 0.01	Apigenin	[6,11]
15	unknown	24.09	290	719, 359	-	-	-

Values are expressed as the mean ± S.D. (*n* = 3).

**Table 3:** Bioactive compounds and antioxidant properties of *D. viscosa* leaves.

Samples	Extract Yield (%)	Polyphenols (mg GAE/g dw)	Flavonoids (mg QE/g dw)	Reducing Power(mg AAE/g dw)	Antioxidant Properties (IC_50_ Values; mg/mL)		
					DPPH	ABTS	Metal Chelating
**AE**	17.97	144.70 ± 4.33 ^a^	78.89 ± 2.43 ^a^	659.44 ± 14.71 ^a^	0.12 ± 0.004 ^a^	0.41 ± 0.01 ^a^	2.39 ± 0.31 ^a^
**ME**	14.2	212.44 ± 13.48 ^a^	123.05 ± 2.87 ^b^	944.19 ± 28.44 ^a^	0.08 ± 0.004 ^b^	0.22 ± 0.03 ^b^	0.51 ± 0.03 ^b^

All the values are mean ± standard deviation. **GAE**: gallic acid equivalents. **AE**: aqueous extract. **ME**: methanolic extract. **QE**: quercetin equivalents. **AAE**: ascorbic acid equivalent. **IC_50_**: The extract concentration provides 50% inhibition. **DPPH:** 2,2-diphenyl-1-picrylhydrazyl. **ABTS:** 2,2′-azino-bis (3ethylbenzothiazoline-6-sulphonic acid). **dw:** dry weight. Different letters indicate significant differences (*p* < 0.05) within conditions according to the two-tailed Student’s *t*-test.

**Table 4:** Inhibition results of *D. viscosa* leaf extracts on both α-amylase and α-glucosidase enzymes.

	IC50 (mg/mL)	
	α-amylase	α-glucosidase
***D. viscosa* leaves**	1.381 ± 0.085 ^a^	0.118 ± 0.02 ^a^
**Acarbose**	0.046 ± 0.001 ^b^	0.329 ± 0.041 ^b^

Values are means ± standard deviation. Different letters in the same column indicate significant differences (*p* < 0.05) within conditions according to the two-tailed Student’s *t*-test.

**Table 5:** The IC_50_ values of cytotoxic activity against MDA-MB-468, MCF-7, and PBMCs and % of viability on PBMCs at different concentrations of the extract and Cisplatin (CisP). Cells were treated with methanolic extracts from *D. viscosa* leaves.

Sample Tested		IC_50_ (µg/mL) Values of Cytotoxity against Tumor Cells		% of Viability in PBMCs		
	MCF-7	MDA-MB-468	PBMCs	Concentration (µg/mL)		
				12.5	3.125	0.78125
*D. viscosa leaves*	2.75 ± 1.2 ^a^	20.43 ± 2.99 ^a^	>50 ^a^	88.54 ± 7.16 ^a^	106.15 ± 4.28 ^a^	256.98 ± 7.6 ^a^
CisP	0.20 ± 0.0 ^c^	2.20 ± 0.40 ^c^	0.27 ^b^	16.08 ± 3.39 ^b^	30.08 ± 3.58 ^b^	37.96 ± 3.44 ^b^

Each value represents the mean ± standard deviation of three independent replicates. Different letters in the same column indicate significant differences *(p* < 0.05) within conditions according to the two-tailed Student’s *t*-test.

**Table 6:** Antimicrobial activity of *D. viscosa* L. leaf extract.

Bacterial Strains	Inhibition Zone Diameter (mm)		Concentration of *D. viscosa* L. (mg/mL)	
	*D. viscosa* L.	*Gentamicine*	MIC	MBC
***Proteus mirabilis***	11.3 ± 0.6	18.7 ± 0.6	6.79 ± 0.39 × 10^−17^	>25
***Bacillus subtilis***	10.3 ± 0.6	19.7 ± 0.6	1.56 ± 2.72 × 10^−16^	>25
***Staphylococcus aureus***	n.e	15 ± 1	-	-
***Pseudomonas aeruginosa***	n.e	n.e	-	-
***Escherichia coli***	n.e	16 ± 1	-	-

The diameter of the inhibition zones (mm), including the diameter of the disc (6 mm), is given as mean ± SD of triplicate experiments. **MIC:** Minimal inhibitory. **MBC:** Minimal bactericidal. **n.e:** no effect.

## Data Availability

Not applicable.

## Sample Availability

Samples of the compounds are available from the authors.
